# Wnt signaling preserves progenitor cell multipotency during adipose tissue development

**DOI:** 10.1038/s42255-023-00813-y

**Published:** 2023-06-19

**Authors:** Zinger Yang Loureiro, Shannon Joyce, Tiffany DeSouza, Javier Solivan-Rivera, Anand Desai, Pantos Skritakis, Qin Yang, Rachel Ziegler, Denise Zhong, Tammy T. Nguyen, Ormond A. MacDougald, Silvia Corvera

**Affiliations:** 1 Morningside Graduate School of Biomedical Sciences, University of Massachusetts Chan Medical School, Worcester, MA USA; 2 Program in Molecular Medicine, University of Massachusetts Chan Medical School, Worcester, MA USA; 3 Division of Vascular Surgery, Department of Surgery, UMass Memorial Medical Center, Worcester, MA USA; 4 Diabetes Center of Excellence, University of Massachusetts Chan Medical School, Worcester, MA USA; 5 Department of Molecular and Integrative Physiology, University of Michigan Medical School, Ann Arbor, MI USA; 6 Division of Metabolism, Endocrinology, and Diabetes, Department of Internal Medicine, University of Michigan Medical School, Ann Arbor, MI USA

**Keywords:** Differentiation, Molecular medicine, Fat metabolism

## Abstract

Mesenchymal stem/progenitor cells are essential for tissue development and repair throughout life, but how they are maintained under chronic differentiation pressure is not known. Using single-cell transcriptomics of human progenitor cells we find that adipose differentiation stimuli elicit two cellular trajectories: one toward mature adipocytes and another toward a pool of non-differentiated cells that maintain progenitor characteristics. These cells are induced by transient Wnt pathway activation and express numerous extracellular matrix genes and are therefore named structural Wnt-regulated adipose tissue cells. We find that the genetic signature of structural Wnt-regulated adipose tissue cells is present in adult human adipose tissue and adipose tissue developed from human progenitor cells in mice. Our results suggest a mechanism whereby adipose differentiation occurs concurrently with the maintenance of a mesenchymal progenitor cell pool, ensuring tissue development, repair and appropriate metabolic control over the lifetime.

## Abstract (web summary)

Single-cell transcriptomic analysis of human multipotent progenitor cells reveals that upon adipogenic stimulation, Wnt signaling regulates the generation of functional multipotent mesenchymal progenitors, named structural Wnt-regulated adipose tissue resident cells, which maintain the progenitor pool.

## Main

Adult somatic tissues contain specialized cells that define organ- and tissue-specific functions. Replacement of these specialized cells when damaged or dead is essential for continuous tissue and organ function throughout the lifetime and depends on the availability of multipotent stem/progenitor cells capable of differentiating into characteristic cell types. The properties of progenitor cells in epithelial tissues, including skin and intestine and in blood, have been well characterized^1–3^, but how mesenchymal progenitor cells, which are involved in the development of bone, cartilage, muscle and adipose tissues are maintained is less clear.

The mechanisms involved in maintaining an adequate pool of multipotent mesenchymal progenitors are particularly intriguing in the context of obesity, given that a large proportion of these progenitor cells are located in adipose tissue^4,5^. Adipose tissue has the ability to expand massively in adults and in cases of severe obesity, over 50% of body mass can consist of adipose tissue^6^. How the pool of multipotent mesenchymal progenitors is sustained under such conditions of chronic differentiation pressure into the adipocyte fate is not known.

Insights into mechanisms underlying adipocyte differentiation have been obtained primarily in mouse models^7^. Two stages of murine adipocyte formation have been defined. The first is the determination phase, during which multipotent mesenchymal progenitor cells become committed to pre-adipocytes and lose the ability to differentiate into other cell types. The second phase consists of terminal differentiation, during which committed pre-adipocytes express genes for lipid transport and synthesis, form specialized lipid droplets and secrete adipocyte-specific cytokines such as adiponectin. The mechanisms underlying terminal differentiation are better understood and include the sequential expression of members of the CCAAT*/*enhancer binding protein (C/EBP), peroxisome proliferator-activated receptor (PPAR) families and the adipocyte determination and differentiation factor-1/sterol response element binding protein 1c (ADD1/SREBP1c). Enforced expression of Wnt10b, which signals through the canonical Wnt pathway, blocks expression of PPAR-γ and C/EBP*-*α and thereby inhibits terminal differentiation of pre-adipocytes^8–10^.

Much less is known about the determination phase by which multipotent mesenchymal progenitors become committed pre-adipocytes, largely due to the lack of suitable cell models; however, a role for Wnt signaling in pre-adipocyte determination is supported by the finding that *Wnt10b*-null mice display a progressive loss of adipogenic and osteogenic progenitors and premature adipogenesis and osteogenesis^11^. While the mechanisms that control human adipocyte development are less well understood, there is a genetic association between *TCF7L2*, a key effector of the Wnt signaling pathway and type 2 diabetes^12–14^ and between *WNT10B* and the development of obesity^15^, consistent with an important role for Wnt signaling in human adipose tissue development.

Previous work has shown that mesenchymal progenitor cells reside in close association with the microvasculature^16–21^ and we have previously reported that culture conditions that promote angiogenesis also promote the proliferation of multipotent mesenchymal progenitor cells^22,23^ which can give rise to multiple human adipocyte subtypes^24^. Here we sought to leverage this technology to investigate how human adipocytes develop from multipotent progenitor cells, by applying single-cell transcriptomics at key stages of differentiation. We find that rapidly upon adipogenic stimulation, human multipotent mesenchymal progenitor cells split into two well defined developmental trajectories; one trajectory leads to the differentiated adipocyte fate, but another generates a pool of cells that can regain proliferative capacity and the ability to differentiate into multiple lineages, including chondro- and osteogenic fates. This multipotent cell pool is characterized by expression of genes for structural, extracellular matrix proteins and by expression of Wnt target genes. Functional studies indicate the relative distribution of cells among these two trajectories is controlled by canonical Wnt signaling. These results reveal a mechanism, elicited under conditions of strong differentiation pressure, that generates a pool of cells that can regenerate functional multipotent mesenchymal progenitors (structural Wnt-regulated adipose tissue (SWAT) cells). This mechanism potentially explains how human mesenchymal tissues can be maintained and repaired throughout the lifetime.

## Results

### Acute transcriptional remodeling upon adipogenic stimulation

**Fig. 1:**
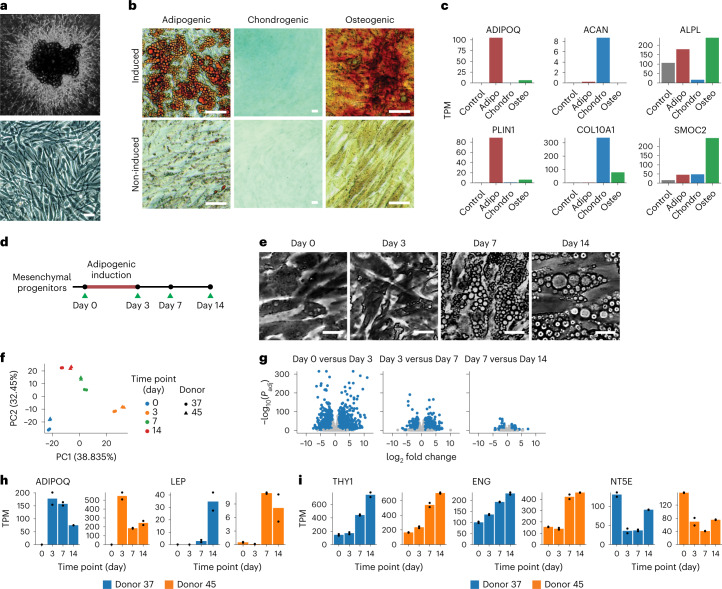
Dynamic transcriptomic changes in multipotent mesenchymal progenitors from human adipose tissue undergoing adipogenic differentiation. **a**, Mesenchymal progenitor cells expanded from adipose tissue explants in three-dimensional culture (top), plated and grown to confluence in two-dimensional culture dishes (bottom; scale bar, 50 μm). **b**, Images of progenitors induced toward adipogenic, chondrogenic or osteogenic cell fates for 10 d. Adipogenic-induced cells were stained with Oil Red O, chondrogenic-induced cells with Alcian blue 8GX and osteogenic-induced cells with Alizarin red S. Scale bar, 50 μm. **c**, Marker genes for progenitors differentiated toward adipogenic, chondrogenic and osteogenic lineages identified using their transcriptomic profile. Bars are means of technical replicates from *n* = 1 wells subjected to the indicated differentiation cocktails. TPM, transcripts per million. **d**, Schematic of the adipogenesis time-course study. **e**, Representative images of mesenchymal cells induced toward adipogenic fate for 0, 3, 7 and 14 d. Scale bar, 50 μm. **f**, Scatter-plot of the first two principal components of bulk RNA-seq results from two independent cultures, each from two independent donors, separately expanded and used to obtain RNA at 0, 3, 7 or 14 d after adipogenic induction. PCA was performed on the expression of the top 1,000 most variable genes across all *n* = 16 samples. **g**, Volcano plots of the differential gene expression analysis results between consecutive time points. **h**,**i**, Time courses of *ADIPOQ* and *LEP* and of mesenchymal progenitor markers *THY1* (*CD90*), *ENG* (*CD105*) and *NT5E* (*CD73*).

We used a previously described method to generate multipotent progenitor cells from human adult adipose tissues^24^. All procedures were conducted in accordance with the UMass Chan Institutional Review Board ID 14734_13. Briefly, small fragments of subcutaneous adipose tissue destined to be discarded from individuals undergoing elective panniculectomy surgery were embedded in Matrigel within 6 h of surgery. After 14 d in culture, extensive growth of capillary sprouts was observed (Fig. 1a). Sprouts were digested using dispase and collagenase type I and plated in plastic culture dishes, where attached cells adopted a fibroblastic homogenous phenotype characteristic of mesenchymal progenitor cells (Fig. 1a). After two passages, cells were frozen for further studies.

**Extended Data Fig. 1:**
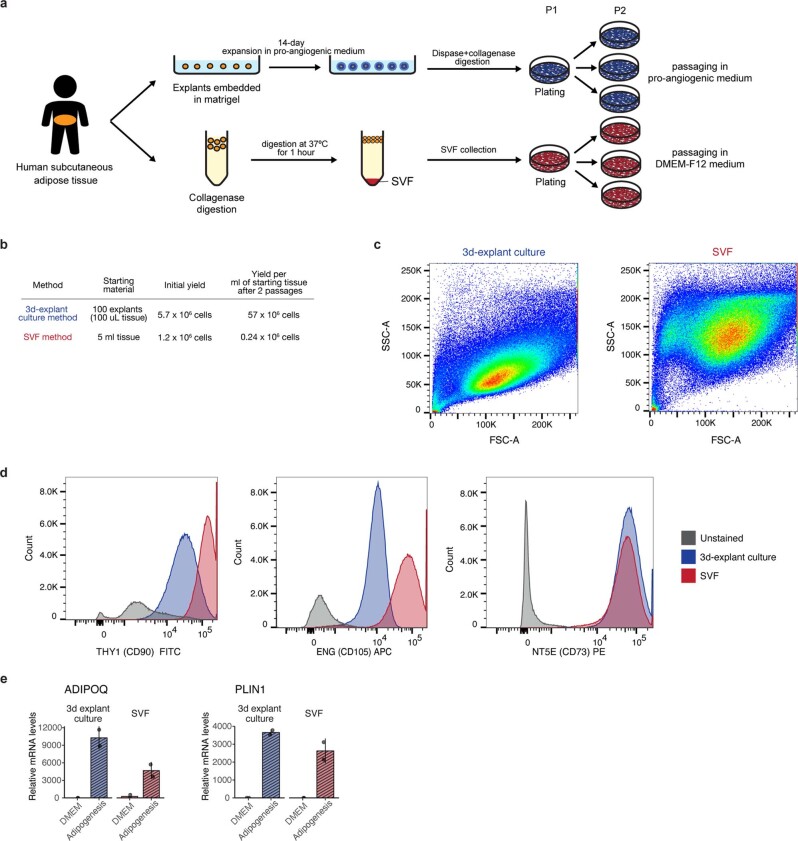
Comparison of 3-dimensional culture-derived progenitor cells and cells obtained from the stromal vascular fraction. **a**, Schematic of the two methods for obtaining progenitors. **b**, Progenitor cell yields from the two methods using adipose tissue from the same donor (Donor 72). **c**, Forward and side-scatter plots of passage 3 progenitors harvested from the two methods, assessed by flow cytometry. **d**, Flow cytometry analysis of mesenchymal progenitor marker levels in passage 3 cells obtained from the two methods. **e**, qRT-PCR assessment of adipocyte markers *ADIPOQ* and *PLIN1* mRNA levels in passage 3 cells collected from the two methods after 7-day of adipogenic induction. Data are shown as mean ± SD. Each dot represents a data point from a biological replicate. Source data

We compared cells obtained by this three-dimensional culture method with the traditional method to obtain progenitor cells from the stromal vascular fraction (SVF) obtained by collagenase digestion of excised tissue (Extended Data Fig. 1a,b). A greater degree of side scatter indicated more heterogeneity in cells obtained by collagenase digestion after plating and passaging, compared to cells expanded in Matrigel (Extended Data Fig. 1c). Both populations displayed cell surface markers THY1 (CD90), ENG (CD105) and NT5E (CD73) (Extended Data Fig. 1d), which define canonical mesenchymal progenitor cell identity^25^ and both populations displayed adipogenic potential as assessed by expression of adiponectin (*ADIPOQ*), an adipocyte-specific cytokine and perilipin 1 (*PLIN1*), an adipocyte lipid droplet-associated protein, following adipogenic induction (Extended Data Fig. 1e). A clear advantage of the three-dimensional culture system is the yield of cells per gram of adipose tissue, which is over 100× greater at passage 2 and over 1,000× greater with further (>5) passages. Mesenchymal progenitor cells obtained by three-dimensional culture retain multipotency, as monolayers exposed to adipose, chondro- or osteogenic differentiation medium for 10 d stained positively for neutral lipid, proteoglycan or calcium, respectively (Fig. 1b). Multipotent differentiation was also seen in their gene expression profiles, where genes associated with the adipogenic (*ADIPOQ* and *PLIN1*), chondrogenic (aggrecan (*ACAN*) and collagen type X α1 chain (*COL10A1*)) or osteogenic (alkaline phosphatase (*ALPL*) and osteonectin-related modular calcium-binding protein 2 (*SMOC2*)) lineages were selectively expressed in response to each cocktail (Fig. 1c). Notably, not all cells underwent differentiation upon adipogenic induction, as cells lacking lipid droplets could be detected alongside lipid replete cells (Fig. 1b).

To explore the timeline for commitment of multipotent cells to the adipogenic fate, we conducted bulk RNA-seq on cells from two independent donors at 0, 3, 7 and 14 d following adipogenic induction with a minimal cocktail of insulin, methyl isobutyl xanthine and dexamethasone added to DMEM (Fig. 1d). These represent times before (0 and 3 d) and after (7 and 14 d) visible lipid droplet accumulation (Fig. 1e). Principal-component analysis (PCA) (Fig. 1f) reveals the largest changes occur between 0 and 3 d of exposure with little variance attributable to the tissue donor. The number of differentially expressed genes between 0 and 3 d of differentiation (3,268 genes) is higher than that seen between 3 and 7 d (1,908 genes) or between 7 and 14 d (413 genes) (Fig. 1g), indicating that major transcriptomic events occur before lipid droplet accumulation. Expression of *ADIPOQ* decreases after 3 d (Fig. 1h), indicating that commitment to the adipogenic fate occurs between 0 and 3 d after induction. Additional adipocyte development continues beyond 3 d, as evidenced by increasing expression of leptin (*LEP*), another canonical adipocyte cytokine (Fig. 1h). Unexpectedly, expression of mesenchymal progenitor cell markers *THY1* and *ENG* increased and *NT5E* remained expressed, over the differentiation time course (Fig. 1i).

### Adipogenic induction elicits two distinct fate trajectories

**Fig. 2:**
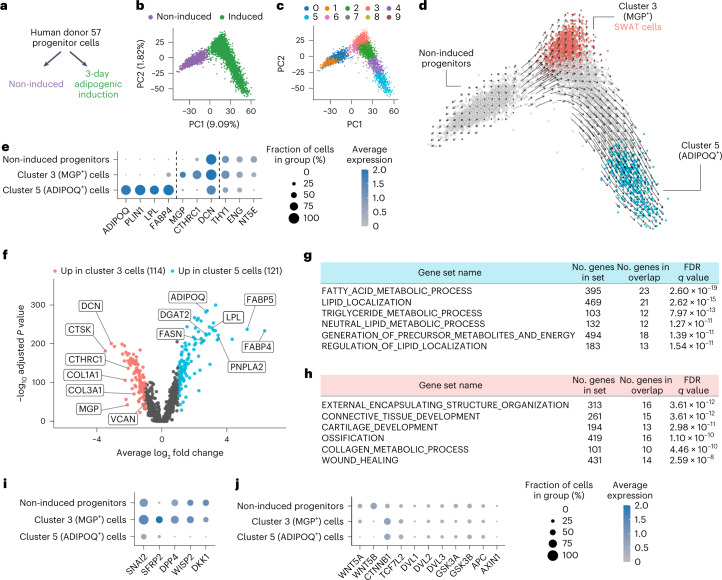
Single-cell RNA-seq of induced adipose progenitors. **a**, Schematic of the early adipogenesis single-cell transcriptomic profiling study. **b**, PCA projection of the single-cell profile of 6,615 cells (3,226 non-induced; 3,430 adipogenic-induced; mean number of genes per cell, 3,382). **c**, Clustering of the single cells with Louvain algorithm. **d**, Inference of developmental trajectory with RNA velocity. Red and blue colored cells represent clusters at the terminals of two projected fate trajectories. **e**, Expression of representative cluster 5 (*ADIPOQ*, *PLIN1*, *LPL* and *FABP4)*, cluster 3 (*MGP*, *CTHRC1* and *DCN*) and mesenchymal progenitor cell marker genes. **f**, Volcano plot comparing cluster 3 and cluster 5 cell gene expression, with differentially expressed genes highlighted. Tested features were limited to genes detected in >25% cells in at least one of the clusters. Differentially expressed genes were defined as those with log_2_ fold change > 1 and adjusted *P* value < 0.001. **g**, Top six significantly enriched gene sets of genes upregulated in cluster 5/*ADIPOQ*^+^ cells. **h**, Top six significantly enriched gene sets of genes upregulated in the cluster 3/SWAT cells. **i**, Dot-plot of the canonical Wnt target genes that were significantly upregulated in cluster 3 cells. **j**, Dot-plot of the detected Wnt ligands and core Wnt pathway members.

**Extended Data Fig. 2:**
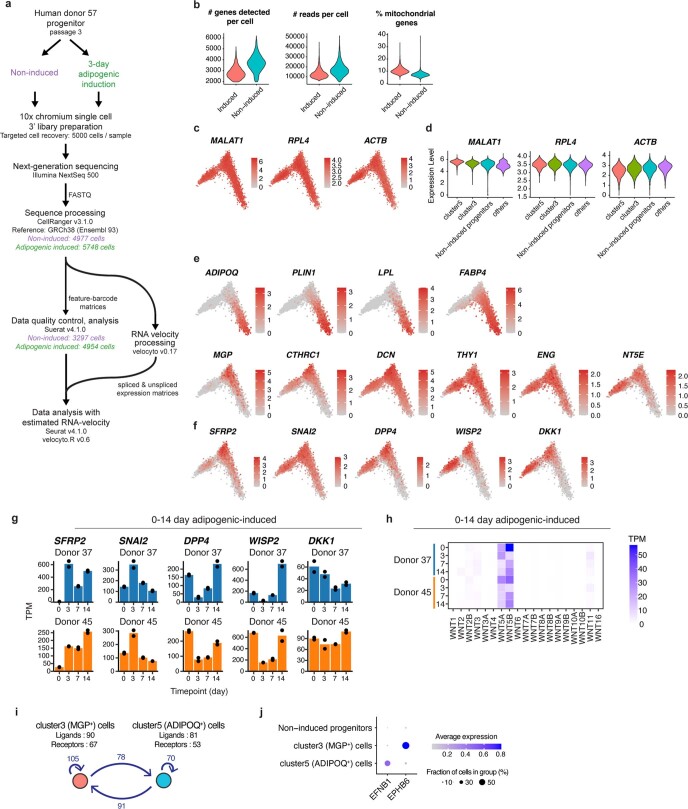
Processing, quality control and data verification of the single-cell RNA-seq profiling of non-induced and 3-day adipogenic-induced progenitors. **a**, Schematic of the sample and bioinformatics processing of the study. **b**, Violin plots of the number of genes/reads detected and estimated percentage of mitochondrial genes in cells that had passed quality control. **c**, Selected housekeeping gene expression in PCA projection. **d**, Violin plots of gene expression distribution of selected housekeeping genes. **e**, Expression of top marker genes for clusters 3 and 5 in PCA projection. **f**, Expression of Wnt target genes in PCA projection. **g**, Gene expression profiles of Wnt target genes upregulated in cluster 3/*MGP*^+^ cell markers in the adipogenesis time course RNA-seq dataset presented in Fig. 1d. **h**, Wnt ligands queried from the adipogenesis time course RNA-Seq dataset presented in Fig. 1d. **i**, Ligand–receptor analysis identifies multiple ligand–receptor interacting pairs between the cluster 3/*MGP*^+^ cells and cluster 5/*ADIPOQ*^+^ cell. **j**, Dot-plot of ephrin-B receptor 6 (*EPHB6*) and ephrin B1 (*EFNB1*) in cluster 3/*MGP*^+^ cells and cluster 5/*ADIPOQ*^+^ cells.

To understand the importance of the simultaneous increase in adipocyte and stem cell markers during the differentiation trajectory, we performed single-cell RNA-seq on two cell pools, one corresponding to multipotent progenitors grown to confluency but not subjected to any differentiation stimuli (non-induced progenitors) and the second corresponding to progenitors exposed to adipogenic medium for 3 d, at which point cells displayed minimal lipid accumulation and remained amenable to microfluidic-based single-cell profiling (Fig. 2a and Extended Data Fig. 2a–d). Projection of cells from these two pools by the top two principal components showed the two populations were non-overlapping (Fig. 2b), indicating that all cells undergo extensive transcriptomic changes upon adipogenic induction. Of note, a broader transcriptomic spectrum is seen in cells subjected to adipogenic induction, as evidenced by the larger distance in the orthogonal first and second principal components.

To gain insight on the nature of the transcriptomic variance, we clustered the single cells into subgroups. Cells subjected to adipogenic induction could be divided into a larger number of clusters, consistent with the PCA (Fig. 2c); however, analysis of developmental trajectory using RNA velocity indicated that, upon induction, progenitors differentiate along two distinct trajectories toward two cell fates (Fig. 2d). The terminal of one trajectory (corresponding to cluster 5) expressed adipocyte-specific genes including *ADIPOQ*, *PLIN1*, lipoprotein lipase (*LPL*) and fatty acid binding protein 4 (*FABP4*) (Fig. 2e and Extended Data Fig. 2e), while the terminal of the other trajectory (cluster 3) did not. Instead, this developmental trajectory was characterized by induction of genes including matrix gla protein (*MGP*) and collagen triple helix repeat containing 1 (*CTHRC1*), which encode for extracellular matrix proteins (Fig. 2e and Extended Data Fig. 2e) and retained expression of genes for mesenchymal stem/progenitor cells markers *THY1*, *NT5E* and *ENG*, as well as for structural proteins seen in non-differentiated cells such as decorin (*DCN*) (Fig. 2e and Extended Data Fig. 2e).

### Cluster 3 cells express canonical Wnt target genes

To further investigate the identity of the non-adipogenic, cluster 3 cells, we conducted differential gene expression analysis comparing cluster 3 and cluster 5 (Fig. 2f). As expected, cluster 5 was enriched in genes associated with adipocyte-specific pathways (Fig. 2g). In contrast, cluster 3 cells were enriched in genes associated with connective tissue development, mostly comprising extracellular matrix proteins (Fig. 2h). Notably, these cells did not express osteocyte (osteocalcin/*BGLAP*, osteopontin/*SPP1*) or chondrocyte (aggrecan/*ACAN*, cartilage collagen/*COL2A1*) markers, suggesting that they do not represent cells undergoing differentiation into an alternative mesenchymal cell lineage. Further analysis of cluster 3 cells revealed that multiple canonical Wnt target genes (*SFRP2, DPP4, DKK1, SNAI2* and *WISP2*) were significantly upregulated (Fig. 2i and Extended Data Fig. 2f). Upstream components of canonical Wnt signaling included genes for ligands (*WNT5A* and *WNT5B)* and core pathway elements including β-catenin (*CTNNB1*) and *TCF7L2* (Fig. 2j). We analyzed bulk RNA-seq transcriptome profiles of cells from additional donors and confirmed that expression of Wnt signaling elements occurs consistently upon induction of differentiation (Extended Data Fig. 2g,h). Moreover, ligand–receptor analysis of potential interactions between *ADIPOQ*^+^ and *MGP*^+^ cells revealed ephrin B1 (*EFNB1*) in cluster 5 and ephrin type-B receptor 6 (*EPHB6*) in cluster 3 cells (Extended Data Fig. 2i,j) which produce repulsive forces in response to Wnt signaling in other cellular contexts^26^. Other significant interactions between cluster 5 and cluster 3 cells included collagen/integrin and other structural extracellular proteins interacting with cognate receptors (Supplementary Table 2). Given the simultaneous induction of genes encoding for structural elements and of genes reflective of Wnt signaling activity, we named cluster 3 cells as SWAT cells.

### SWAT cells retain progenitor features and have high Wnt activity

**Fig. 3:**
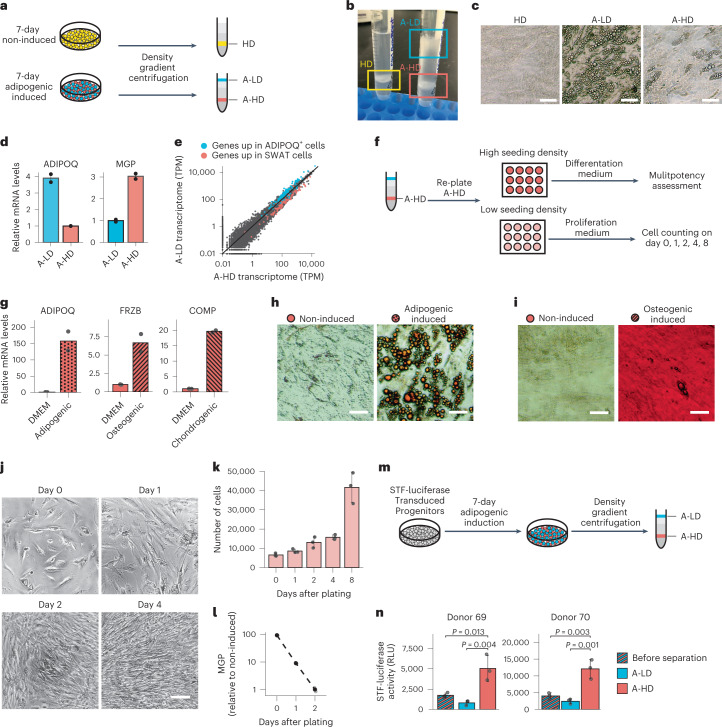
SWAT cells are multipotent mesenchymal progenitors with high canonical Wnt activity. **a**, Schematic of the cell separation assay with Percoll density gradient centrifugation. **b**, Image of cells after density centrifugation. **c**, Separated cell layers from **b**, re-seeded in multi-well plates. Images were taken 72 h after seeding. **d**, qRT–PCR assessment of *ADIPOQ* and *MGP* messenger RNA levels in A-LD and A-HD from 7-d adipogenic-induced cells (*n* = 2). **e**, Scatter-plot of each detected gene’s TPM values between the high- and low-density cells measured by bulk RNA-seq. Genes highlighted in blue were the 121 genes enriched in the cluster 5/*ADIPOQ*^+^ cells, as identified by differential expression analysis of single-cell RNA-seq presented in Fig. 2f and genes highlighted in red were the 114 genes enriched in the cluster 3/SWAT cells. **f**, Schematic of the experiment assessing multipotent and proliferative potential of A-HD cells obtained from 7-d adipogenic-induced cells. For low-seeding-density plating, 10,000 cells were plated per well in a 96-well plate; for high-seeding-density plating, 30,000 cells were plated per well. **g**, mRNA levels of adipogenic (*ADIPOQ*), chondrogenic (*COMP*) and osteogenic (*FRZB*) lineage markers of A-HD after 3-d lineage differentiation (*n* = 2). **h**, Oil Red O staining of A-HD after 10-d adipogenic induction. Scale bar, 50 μm. **i**, Alizarin red S staining of A-HD after 10-d osteogenic induction. Scale bar, 50 μm. **j**, Phase images of A-HD after growth for indicated days in progenitor growth medium. Scale bar, 100 μm. **k**, Cell counts of A-HD after indicated days in progenitor growth medium (*n* = 3). **l**, qRT–PCR assessment of *MGP* mRNA levels in A-HD cells collected after indicated days in progenitor growth medium. **m**, Schematic of cell separation assay with STF luciferase reporter. **n**, STF luciferase levels in A-LD and A-HD cells before and after cell separation (*n* = 3, *P* values are determined by one-way analysis of variance (ANOVA)). RLU, relative light units. All data plots are shown as mean ± s.d. Each dot represents a data point from a biological replicate. Source data

To further investigate the functional characteristics of SWAT cells, we leveraged the fact that accumulation of lipid droplets during adipocyte differentiation decreases cell density. Adipogenic cells could thereby be separated from non-adipogenic cells by centrifugation through Percoll gradients (Fig. 3a). Mesenchymal progenitors that were not induced to differentiate were recovered between the 1.02–1.04 g ml^−1^ density steps (referred to as high-density (HD) cells), whereas cells differentiated for 7 d were recovered in two populations (Fig. 3b), one at the 1.01–1.02 g ml^−1^ (adipogenic culture low-density (A-LD) cells) and another between 1.03–1.04 g ml^−1^ density steps (adipogenic culture high-density (A-HD) cells). Upon replating, A-LD cells contained abundant lipid droplets, while HD and most A-HD cells did not (Fig. 3c). Quantitative PCR with reverse transcription (qRT–PCR) confirmed a significant enrichment of *ADIPOQ* in A-LD cells and of *MGP* in A-HD cells (Fig. 3d). Moreover, bulk RNA-seq confirmed that all cluster 5 markers were enriched in the A-LD cells, whereas SWAT cell markers were enriched in the A-HD cells (Fig. 3e), confirming that the separation approach was effective and allowing further characterization.

**Extended Data Fig. 3:**
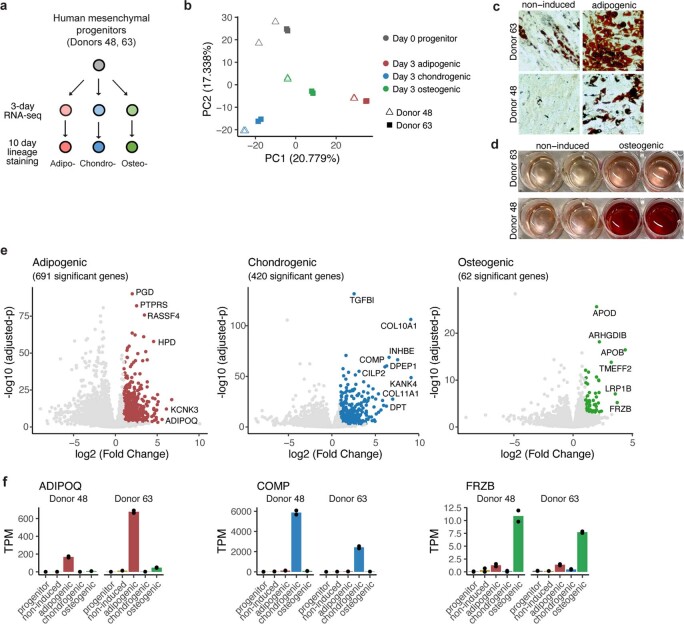
Identification of early osteogenic and chondrogenic lineage marker with multi-lineage time course bulk RNA-seq. **a**, Schematic of the RNA-seq profiling study. **b**, Scatter-plot of the first two principal components of the RNA-seq samples. Principal component analysis was performed on the expression of the top 1000 most variable genes across all samples. **c**, Oil Red O staining of progenitor cells after 10-day adipogenic induction. Scale bars, 50 µm. **d**, Alizarin Red S staining of progenitor cells after 10-day osteogenic induction. **e**, Volcano plots of differential gene expression analysis between cells induced toward the annotated lineage compared to cells induced to other two lineages. Differentially expressed genes were defined as those with log2 fold change > 1 and adjusted *P* value < 0.001. **f**, Gene expression of the most distinctive lineage marker genes, selected based on magnitude and significance in the differential expression analysis as well as specificity. *ADIPOQ*: adiponectin*, COMP*: cartilage oligomeric matrix protein, *FRZB*: frizzled related protein.

To determine whether SWAT cells retain multipotency, A-HD cells obtained after 7 d of adipogenic differentiation were replated and exposed to either adipogenic, osteogenic or chondrogenic differentiation medium. After 3 d, RNA was extracted and expression of early lineage markers previously defined for adipo-, chondro- and osteogenic fates (Extended Data Fig. 3) was measured by qRT–PCR. SWAT cells expressed each of the lineage markers (Fig. 3g) indicating that they can undergo multiple mesenchymal cell fates. Parallel cultures of cells induced with adipogenic or osteogenic cocktails stained after 10 d revealed lipid droplets and calcium aggregates upon adipogenic or osteogenic differentiation, respectively (Fig. 3h,i), verifying differentiation into functional mesenchymal cell lineages. To determine proliferative capacity, A-HD cells were plated at low seeding density and cultured in the medium used to expand multipotent progenitors. Within 24 h, cells began to proliferate and regained the spindle-like fibroblastic morphology characteristic of progenitor cells (Fig. 3j,k). Moreover, *MGP* expression declined in parallel, indicating that cells revert to a low Wnt- activity state resembling the non-induced, *MGP*-negative progenitors (Fig. 3l).

SWAT cells have elevated expression of canonical Wnt target genes, suggestive of Wnt activation. To verify that Wnt signaling is active in SWAT cells, we used the Super TopFlash (STF) reporter^27^, which contains seven *LEF*/*TCF* binding sites controlling expression of firefly luciferase. The STF reporter was transduced into progenitor cells before adipogenic induction and 7 d later cells were separated by Percoll density centrifugation (Fig. 3m). A-HD cells displayed significantly higher reporter activity compared to A-LD cells, confirming that SWAT cells have elevated canonical Wnt activity.

### Wnt controls balance between adipogenic and SWAT fates

**Fig. 4:**
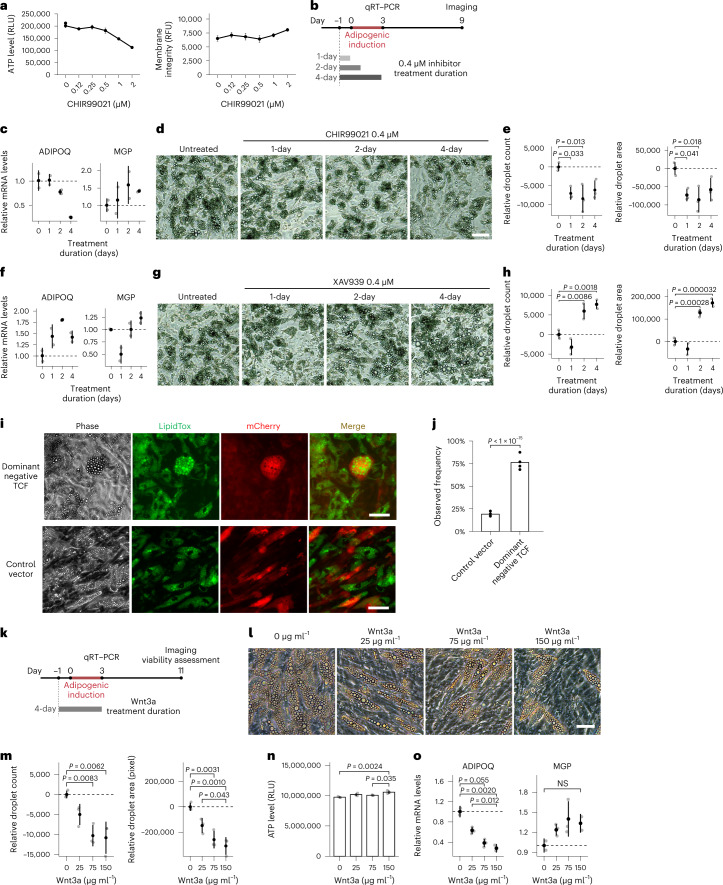
Canonical Wnt signaling controls cell fate balance between adipogenic differentiation and progenitor maintenance. **a**, ATP levels (left) and membrane permeability (right) in 10-d adipogenic-induced cells exposed to CHIR99021 (*n* = 3). **b**, Schematic of the assay assessing effects of low dose, acute Wnt perturbation. **c**, *ADIPOQ* and *MGP* mRNA in cells treated with 0.4 μM CHIR99021 as in **b**, after 3 d of differentiation (*n* = 2). **d**, Images of cells treated with 0.4 μM CHIR99021 as in **b**, after 9 d of differentiation. **e**, Lipid droplet quantification of cells in (**c**) (*n* = 3). **f**, *ADIPOQ* and *MGP* mRNA levels of cells treated with 0.4 μM XAV939 after 3 d of differentiation (*n* = 2). **g**, Images of cells treated with 0.4 μM XAV939 as indicated in **b**, after 9 d of differentiation. **h**, Lipid droplet quantification of cells in **g** (*n* = 3). **i**, Fluorescence microscopy images of 7-d adipogenic-induced cells transduced with vectors expressing control or dominant-negative TCF and an mCherry reporter 72 h before adipogenic induction. Cells were stained with LipidTOX Green neutral lipid stain before imaging. **j**, Frequency of adipogenesis among the transduced, mCherry-positive cells. Quantitation represents four independent experiments with cells from four separate donors. A 2 × 2 contingency table was built for the quantification from each experiment (Extended Data Fig. 4b,d,f,h) and the *P* value was determined by two-sided Cochran–Mantel–Haenszel chi-squared test. **k**, Schematic of the assay to assess effects of acute canonical Wnt activation with recombinant Wnt3a during initial days of adipogenic induction. **l**, Images of cells treated with Wnt3a per described in **k**. **m**, Lipid droplet quantitation of cells in **l** (*n* = 3). **n**, ATP level of cells in **l** measured immediately after live-cell imaging (*n* = 3). **o**. *ADIPOQ* and *MGP* mRNA levels of cells treated with Wnt3a measured by qRT–PCR after 3 d of differentiation (*n* = 2). All data plots in this figure are shown as means ± s.d. Each dot represents a data point from a biological replicate unless stated otherwise. One-way ANOVA is used for statistical analysis unless stated otherwise. Scale bars, 50 μm. NS, not significant. Source data

The observation of high Wnt activity and Wnt target gene expression in SWAT cells raised the possibility that Wnt signaling plays a functional role in the maintenance of multipotent progenitor cells. To test this possibility, we examined the effects of perturbing canonical Wnt signaling during adipogenic induction. CHIR99021 acts by inhibiting GSK3 and preventing phosphorylation-induced degradation of β-catenin, thus enhancing Wnt signaling. In preliminary experiments (Fig. 4a), we found that at concentrations above 0.5 μM CHIR99021 resulted in a decrease in ATP levels and an increase in membrane permeability, suggestive of compromised cell viability. Accordingly, we used a lower dose (0.4 μM) and restricted exposure to the days during which fate determination occurs (days −1 to 3 of adipogenic induction) (Fig. 4b). Exposure to the inhibitor for only 2 d (from −1 to +1 d of induction) resulted in a decreased expression of *ADIPOQ* and reciprocal increase in *MGP* expression in response to adipogenic induction (Fig. 4c), although *MGP* levels were more variable. These effects were durable, as the number and size of lipid droplets measured 9 d after induction were decreased even when exposure to CHIR99021 was restricted to 1 d before addition of the adipogenic cocktail (Fig. 4d).

To test whether suppression of canonical Wnt signaling would have the converse effect, we tested the effects of XAV939, a tankyrase inhibitor that stabilizes AXIN and promotes β-catenin degradation^28^. An increase in *ADIPOQ* levels at day 3 (Fig. 4f) and an increase in lipid droplet number and size at day 9 (Fig. 4g,h) were seen in response to 1–2 d of exposure to the Wnt inhibitor. Notably, *MGP* expression level changes in response to CHIR990021 or XAV939 trended opposite in direction to expression level changes in *ADIPOQ* (Fig. 4c,f).

**Extended Data Fig. 4:**
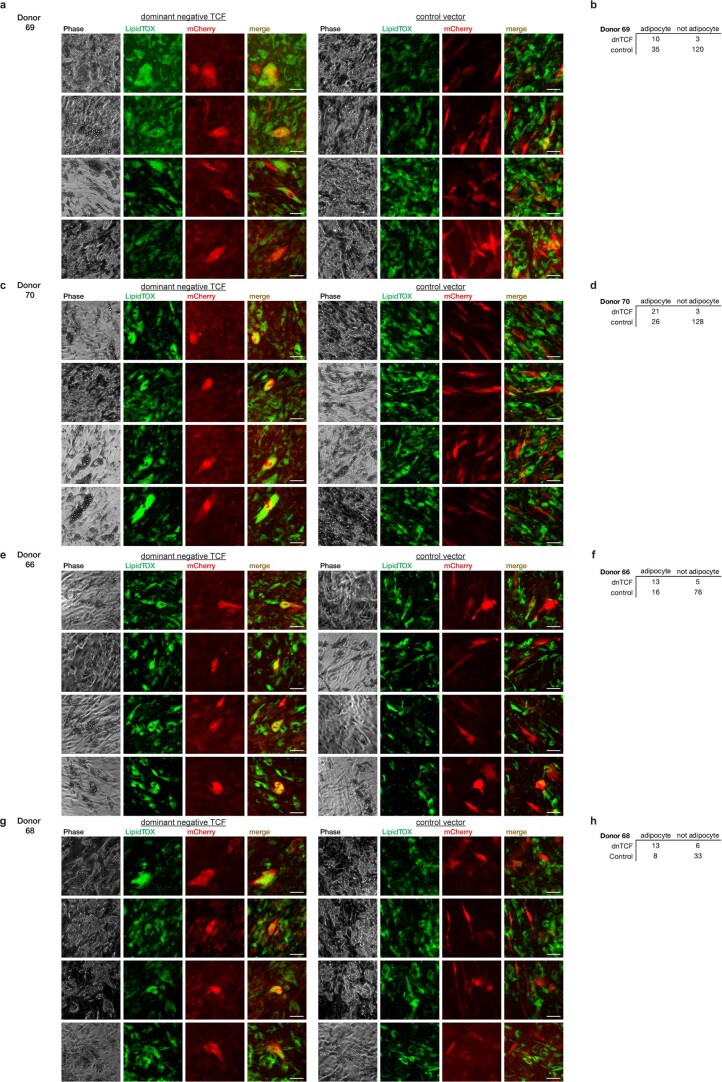
Dominant negative *TCF* expressing progenitors have adipogenic fate preference. **a, c, e, g**, Representative fluorescence microscopy images of 7-day adipogenic-induced cells from 4 separate donors, transduced with lentiviral vectors expressing control or dominant-negative *TCF* and mCherry reporter. The transduction was performed 72 hours prior to adipogenic induction. Cells were stained with LipidTOX green neutral lipid stain prior to imaging. Scale bars, 50 μm. **b, d, f, h**, Quantification of adipocytes and non-adipocytes (inferred by positive and negative LipidTOX signal) among the transduced cells (inferred by positive mCherry signal).

As GSK3 and tankyrase have substrates beyond β-catenin and AXIN, respectively^28–30^, we sought to verify our findings by perturbing Wnt signaling with orthogonal genetic approaches. We leveraged two Wnt pathway modulating reagents, a dominant-negative *TCF* construct (dnTCF) and a constitutively active β-catenin (dpBCat)^31^. Expression of dnTCF in adipogenic-induced human mesenchymal progenitor cells gave rise to adipocytes that seemed larger in comparison to the neighboring mCherry-negative adipocytes (Fig. 4i) and in comparison to those transduced with control mCherry vector (Fig. 4i). To quantitatively assess whether dnTCF transduction promoted the adipogenic fate, we quantified the number of mCherry-positive cells containing lipid droplets in cultures transduced with dnTCF or control vector. Quantifying multiple fields of cells from four independent donors (Extended Data Fig. 4), 68–88% of mCherry-positive cells observed after transduction with dnTCF contained lipid droplets, compared to only 17–23% of mCherry-positive cells transduced with control vector (Fig. 4j), indicating that inhibition of Wnt signaling promotes the adipogenic fate.

Despite robust expression of in HEK293 cells, we were unable to find mCherry-positive cells upon transduction of mesenchymal progenitors with dpBCat, suggesting that chronic Wnt activation or activation above physiological levels is toxic to these cells. As an alternative orthogonal approach, we stimulated progenitors with recombinant Wnt3a protein, which elicits canonical Wnt signaling through broad interactions with multiple receptors^32^, during days −1 to 3 of adipogenic induction (Fig. 4k). This early exposure to Wnt3a resulted in a dose-dependent decrease in the number and size of lipid droplets measured at day 11 post-induction (Fig. 4l,m), with no impact on cell viability (Fig. 4n). Decreased expression of *ADIPOQ* and an opposite trend in *MGP* expression were also observed by RT–PCR (Fig. 4o), consistent with results obtained with Wnt signaling activation upon pharmacological inhibition of GSK3 (Fig. 4c–e). Taken together, our results suggest that human mesenchymal progenitor cells are highly sensitive to canonical Wnt signaling and subtle activation of this pathway is sufficient to shift cell fate toward maintenance of a non-differentiated progenitor pool.

### Wnt activity is induced before cell fate determination

**Fig. 5:**
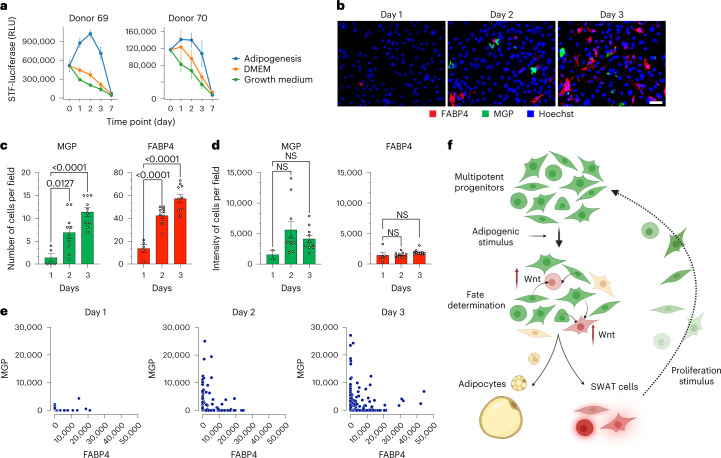
Early canonical Wnt signaling induced by adipogenic stimuli coincides with progenitor cell fate. **a**, Time-course measurement of canonical Wnt activity upon adipogenic induction on two separate donor cells expressing STF luciferase reporter. **b**, Representative RNAscope images of 1-, 2- and 3-d adipogenic-induced cells probed for *FABP4* (red), *MGP* (green) and 4,6-diamidino-2-phenylindole (DAPI) (blue). Scale bar, 50 μm. **c**,**d**, Quantitation of *MGP-* and *FABP4*-positive cell number (**c**) and total signal intensity (**d**) from RNAscope images of 1-, 2- and 3-d adipogenic-induced cells. Each data point represents quantification from one distinct field on the coverslip (*n* = 10). Data are shown as mean ± s.d. One-way ANOVA was used for statistical analysis. **e**, Scatter-plots of *FABP4* and *MGP* intensity of individual cells. Each dot represents one cell. **f**, Model depicting potential sequence of events upon adipogenic induction, consistent with data presented. Canonical Wnt activity is upregulated early upon receiving adipogenic signal, followed by fate determination leading to emergence of SWAT cells and early adipocytes. SWAT cells can regain proliferative capacity and multipotency. Source data

The results above indicate that early, transient perturbation in Wnt signaling is sufficient to result in long-term changes in adipose differentiation. To determine whether Wnt activation actually occurs early after adipogenic induction, we measured Wnt activity over time using the STF luciferase reporter in cells maintained under proliferating conditions, under non-proliferating conditions in the absence of adipogenic induction and in response to adipogenic induction. Induction of Wnt activity was seen only upon adipogenic induction, appearing within 24 h and peaking within 48 h (Fig. 5a). In parallel, we observed the emergence of two cell fates, evidenced by the mutually exclusive expression of *MGP* and *FABP4* mRNA detected by RNAscope (Fig. 5b). Expression was undetectable in non-induced cells but was detectable at day 2. Between days 2 and 3, the number of cells expressing either *MGP* or *FABP4* increased significantly (Fig. 5b,c), but the intensity of each transcript per cell did not (Fig. 5b,d), indicating a rapid commitment of cells to each fate concomitantly with induction of Wnt signaling. Quantification of signal intensities of each transcript per cell within multiple fields confirmed that expression was largely mutually exclusive and that more cells expressed *MGP* or *FABP4* over time (Fig. 5e). Together our results are consistent with a model whereby adipogenic stimulation of multipotent mesenchymal progenitors elicits Wnt signaling, which generates a population of cells (SWAT cells) that are capable of proliferating and replenishing the progenitor pool (Fig. 5f).

### SWAT cells in adipose tissue in vivo

**Fig. 6:**
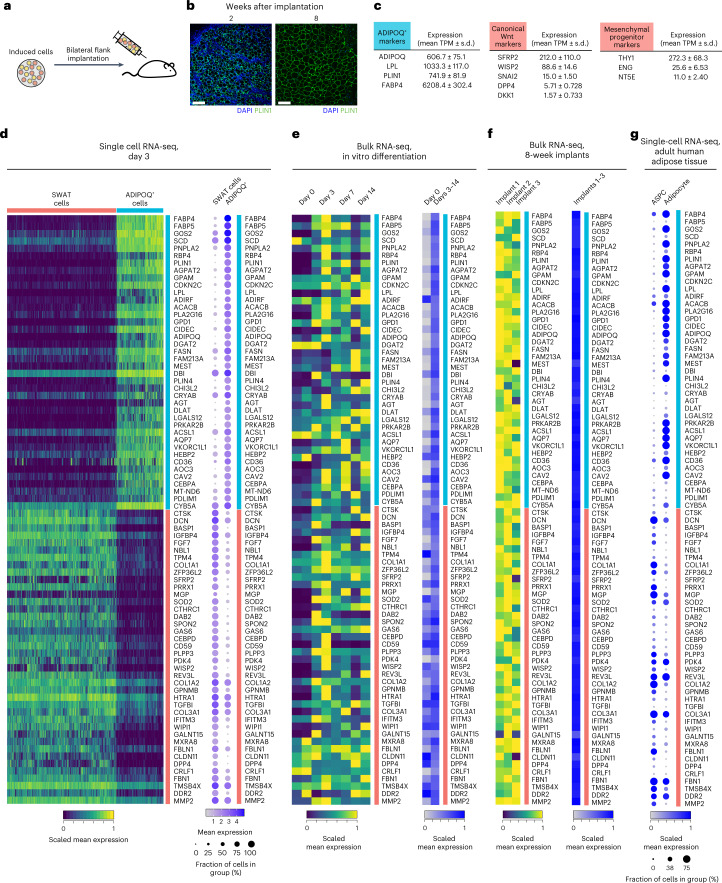
Evidence for SWAT cells in developing and adult adipose tissue in vivo. **a**, Schematic of the human adipogenic-induced progenitor mouse implantation model. **b**, Histological sections of implants 2 weeks (left) and 8 weeks (right) after injection. Scale bar, 150 μm. **c**, mRNA levels of the *ADIPOQ*^+^ cell markers and canonical Wnt target genes in the implants measured by bulk RNA-seq. Results represent summary of three implants from three mice. **d**, Heat maps of individual cells and summary dot-plot of gene expressions of the top 40 differentially expressed gene from SWAT and *ADIPOQ*^+^ cells, derived from the single-cell dataset described in Fig. 2. **e**, Gene expression of the top 40 marker gene of SWAT and *ADIPOQ*^+^ cells from the adipogenesis time course bulk RNA-seq dataset described in Fig. 1d, presented as individual samples (left) or summarized by adipogenic induction status (right). **f**, Heat maps of expression of the top 40 differentially expressed gene of SWAT and *ADIPOQ*^+^ cells from the human reads of the implant dataset presented as individual samples (left) or summarized (right). **g**, Dot-plot of gene expression of the top 40 marker gene of SWAT and *ADIPOQ*^+^ cells in the published single-cell/single-nuclei adipose tissue transcriptome of adult human ages 29–73 years published by Emont et al.^34^.

We next sought to determine whether SWAT cells are present during human adipose tissue development in vivo. We leveraged a model in which human mesenchymal progenitor cells are implanted into immunocompromised mice (Fig. 6a) and generate a functional human/mouse hybrid adipose depot (Fig. 6b)^33^. Analysis of human transcript-specific reads in adipose tissue formed 8 weeks after implantation revealed expected expression of human adipocyte genes, as well as expression of mesenchymal progenitor markers and canonical Wnt target genes (Fig. 6c) indicating that a pool of multipotent progenitors was actively maintained in an in vivo environment. We further selected the top 40 genes differentially expressed between SWAT and *ADIPOQ*^+^ cells to generate a signature for each cell type (Fig. 6d) and analyzed these signatures both during adipocyte differentiation in vitro (Fig. 6e) and during adipose tissue formation in vivo (Fig. 6f). We find that SWAT and *ADIPOQ*^*+*^ cell signatures are rapidly induced and persist both in vitro and in vivo after tissue development.

**Extended Data Fig. 5:**
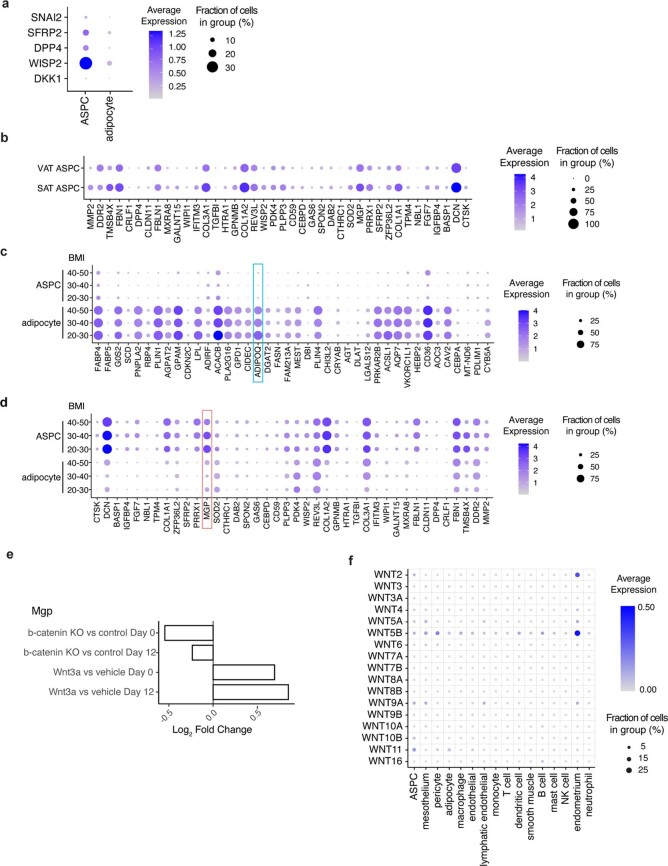
SWAT cell signatures human adipose tissue and mouse progenitors. The Emont, et al. dataset^35^ queried for: **a**, Canonical Wnt target genes expression. **b**, Dot-plot of the top 40 SWAT cell marker genes filtered by human adipose tissue depot. **c**, **d** Dot plots of top 40 *ADIPOQ*^+^ cell (**c**) and SWAT cell (**d**) marker genes, grouped by cell type (ASPC, Adipocyte) and body mass index (BMI). **e**, Bar graph of *Mgp* log_2_ fold change values from four separate differential expression analyses of primary mouse mesenchymal progenitors isolated directly from *β-Catenin*^fl/fl^ mice from Bagchi, et al. dataset^8^. Non-adipogenic-induced progenitor (annotated as ‘Day 0’) or progenitors underwent 12-day adipogenic induction (annotated as ‘Day 12’) were harvested for RNA-seq under Wnt perturbations: Day 0 or Day 12 cells were either induced for β-catenin knockout (annotated as ‘KO’) or were treated with 20 ng/ml recombinant Wnt3a or vehicle for 4 hours before harvest (*n* = 4 per group). **f**, Dot-plot of Wnt ligand expression in all human adipose tissue cell types, from the Emont, et al. dataset^35^. Source data

To determine whether the SWAT and the *ADIPOQ*^+^ cell populations are present in human adult adipose tissue, we leveraged an atlas of single-cell and single-nuclei transcriptomes provided by Emont et al.^34^. We find a clear signature of SWAT cells in mesenchymal progenitor cells (denoted as ASPCs) and of *ADIPOQ*^+^ cells in mature adipocytes (Fig. 6e), indicating that SWAT cells are maintained in adult human adipose tissue. Moreover, expression of Wnt target genes characteristic of SWAT cell genes is seen in ASPCs but is negligible in mature adipocytes (Extended Data Fig. 5a). Notably, an overall higher level of SWAT cell marker genes is seen in ASPCs of subcutaneous adipose tissue (SAT) compared to visceral adipose tissue (Extended Data Fig. 5b), suggesting that cell fate determination dynamics are depot dependent. Moreover, a decrease in both *MGP* and *ADIPOQ* is seen with increasing body mass index (Extended Data Fig. 5c,d), suggesting that chronic adipogenic stimulation might lead to a decreased abundance of progenitor cells capable of adipocyte differentiation. The Wnt-induced generation of SWAT cells in vivo is also supported by RNA-seq results from primary mouse mesenchymal progenitors derived from adipose tissue-specific β-catenin knockout mice^8^. These cells display decreased amount of *MGP*, which is increased in response to Wnt3a treatment (Extended Data Fig. 5e), implying an association of canonical Wnt signaling and SWAT cell development in mouse adipose tissue.

## Discussion

Multipotent mesenchymal progenitor cells are required throughout an individual’s lifetime to renew and repair multiple tissues. In this study, we find that human multipotent mesenchymal progenitors, which seem homogenous at single-cell resolution, rapidly diverge toward two developmental trajectories upon adipogenic induction; one trajectory is toward the adipocyte phenotype, whereas the other is toward a non-differentiated state that maintains proliferative capacity and multipotency. Functional studies identify canonical Wnt signaling as the mechanism involved in generating the non-differentiated progenitor pool. Our findings provide a potential mechanism by which mesenchymal progenitor cells are maintained throughout life. Variations in this mechanism, through genetic or environmental causes, could underlie inter-individual differences in tissue repair capacity and, given the essential role of adipocyte function in systemic energy homeostasis, also contribute to metabolic disease risk.

A limitation of our study is that our results were obtained solely from cells originating from human SAT; however, in their accompanying paper, Palani et al. found very similar developmental trajectories in cells derived from different adipose depots, including supraclavicular, perirenal, subcutaneous and visceral adipose depots from multiple donors. Like us, they found that progenitor cells are transcriptionally homogenous, without any visible structure or separation in pseudo time until adipogenic induction, when they separate into two distinct cell fates. They also find that one of the developmental trajectories encodes genes classically associated with adipocyte differentiation, whereas the second is characterized by expression of multiple extracellular matrix structural factors and Wnt pathway developmental genes. *MGP* was also a major identifier of this branch. Accordingly, we named this branch SWAT cells. Together our results support a model in which differentiation stimuli triggers a process that generates both differentiated adipocytes and a reservoir of cells capable of maintaining a multipotent progenitor state.

Adipose tissue development at single-cell resolution has been more extensively studied in mouse models, where adipose tissue turnover is fast, diverse physiological states can be imposed and genetic tracing approaches can be implemented. Understanding human adipose tissue development is more challenging, in large part due to the fact that the turnover rate of adipocytes in adult humans is exceedingly slow^35^ and therefore developmental trajectories cannot be captured at steady state. Indeed, while single-cell/single-nuclei profiles of human adipose tissue cells capture adipocyte progenitors as distinct spatial clusters, their developmental trajectory toward adipocytes is not well captured^34,36^. The ability to generate adipose tissue progenitor cells at scale has enabled us to analyze the earliest stages of human adipocyte development and allowed comparison with single-cell and single-nuclei profiles of adult human adipose tissue to infer physiological relevance. Through meta-analysis of published transcriptomic data from Emont et al.^34^, we provide evidence that SWAT cells are present in ASPCs in human adult adipose tissue and they are likely maintained throughout lifetime. Palani et al. also found that all three cell types (non-induced progenitors, adipogenic and SWAT cells) can be mapped to cell types in multiple single-cell or single-nuclei datasets of human white and brown adipose tissue. Moreover, in a spatial transcriptomic study of adult human adipose tissue, Backdahl et al.^37^ identified *MGP* as a marker of specific progenitor cell sub-populations interspersed in the tissue.

How SWAT cells correspond to mouse adipose tissue progenitors is unclear. Multiple groups have identified progenitor cell subtypes in murine adipose depots^36,38–42^, some of which express multiple extracellular structural proteins and are capable of suppressing adipogenesis^40,43,44^. Notably, one of the anti-adipogenic factors secreted by murine progenitor cells acts through *Lgr4* (ref. ^43^), which plays a major role in enhancing Wnt activity^45^, suggesting that human and mouse adipose tissue progenitor cells share developmental properties. Also of interest is the identification of *Dpp4* as a marker for a highly proliferative, multipotent progenitor population that can give rise to both committed pre-adipocytes and an alternative, adipogenic cell population^41^. In our studies, *DPP4* is present both in non-induced progenitors and in SWAT cells, consistent with its role as a marker of multiple progenitor populations. *Dpp4* has been reported to be a Wnt downstream target gene^46^ modulated through TGF-β signaling. In our study, we find *MGP* as the most representative marker for the SWAT cell population, as the gene is not expressed in non-induced progenitors or in cells undergoing adipogenesis. MGP is a secreted protein with a known role in preventing tissue calcification^47,48^, but how *MGP* is induced and whether it plays a functional role in supporting the development of the SWAT cell population are remaining questions. *Mgp* is expressed in a sub-population of progenitor cells in mouse subcutaneous adipose tissue^34^ and further analysis will be required to define whether *Mgp*^+^ murine progenitor cells are analogous to SWAT cells in their ability to be rapidly induced in response to adipogenic differentiation and to revert to a non-differentiated state.

The unique ability of adipocytes to accumulate lipids in defined large droplets results in their decreasing density and allows their physical separation from cells that do not accumulate lipids. We found that non-lipid laden cells are highly enriched in SWAT cell markers, allowing functional assessment of the SWAT cell population. Once separated from adipocytes, SWAT cells can differentiate into osteogenic, chondrogenic and adipogenic lineages, indicating they retain multipotency. Moreover, SWAT cells rapidly regain proliferative capacity when placed in either microvascular cell growth medium, or as shown by Palani et al. in DMEM/F12. The recovery of proliferative capacity rules out cellular senescence as a reason for the failure of these cells to undergo adipogenic differentiation. Rapidly after induction of proliferation, SWAT cells lose expression of the specific marker *MGP*, suggesting they revert to the phenotype of cells that were never subjected to adipogenic stimulation. Similar conclusions were reached through complementary experiments by Pallani et al., who observed that after induction of proliferation SWAT cells regain expression of genes (*ID1*, *ID3*, *KRT18* and *POSTN*) that are only present in progenitor cells that were not exposed to adipogenic stimulation.

A limitation of our studies is that we have not assessed whether SWAT cells can fully revert to a never-induced progenitor state after sustained proliferation; however, Palani et al. performed full transcriptomic analysis of SWAT cells after exposure to proliferation medium for only 24 h and observed transcriptomes poised between those of SWAT and progenitor cells, indicating a global reversion trajectory. Further experiments comparing the full transcriptomes of SWAT cells before and after sustained proliferation, with the transcriptomes of progenitors that were never exposed to differentiation stimuli will be required to determine how completely these cells reverse to a naive multipotent state. The detailed mechanisms whereby SWAT cells are generated in response to adipogenic stimulation, as well as the mechanisms that determine their subsequent proliferative capacity and reversion to a multipotent progenitor state, are yet to be fully elucidated. These mechanisms could play a vital role in determining key features of adipose tissue, such as its propensity for hyperplasia or hypertrophy and overall depot size and distribution over the lifetime.

Single-cell trajectory analysis and experiments monitoring appearance of *MGP* and *FABP4* mRNAs by RNAscope indicate that SWAT and adipogenic cells are induced rapidly, simultaneously and dichotomously from a transcriptomically homogenous progenitor pool. Moreover, Palani et al. found that single, non-induced cells plated into separate wells of a 96-well multi-well plate develop into both adipogenic and SWAT cells in varied proportions. These results together suggest that SWAT and adipogenic cells arise stochastically from a common progenitor cell. The involvement of Wnt signaling is compatible with a stochastic process, as Wnt signals can instruct cell identities in a signaling strength-dependent manner^49^ and PPAR-γ and Wnt signaling are both antagonistic to each other^50^. Thus, small differences in expression of Wnt ligands or receptors in vicinal cells, together with mutually reinforcing signaling pathways can lead to rapid divergence in cell fate.

Wnt has important roles in stem cell maintenance in multiple tissues and organs^1–3^. Recently, de Winter and Nusse^51^ reviewed existing studies of the role of Wnt in adipogenesis^10^. They noted that inactivation of Wnt signaling is necessary for mesenchymal progenitor lineage commitment and further postulate that Wnt inhibits adipogenesis by promoting mesenchymal progenitor maintenance. Our findings provide direct evidence for that hypothesis, showing that, via induction of SWAT cells, Wnt signaling drives cells toward a progenitor state even in the context of strong adipogenic induction.

Our study is limited by our lack of knowledge of the specific ligands and receptors that elicit Wnt activation upon adipogenic stimulation. The adipogenic cocktail we use consists only of insulin, dexamethasone and methyl isolbutyl xanthine, which induce Wnt signaling within 24 h. Insulin has been reported to stimulate Wnt signaling^52,53^ and understanding this role in the context of glucocorticoid stimulation and phosphodiesterase inhibition will be the subject of future work. This early induction of Wnt is crucial, as pharmacological or genetic modulation restricted to the first 24–48 h following adipogenic induction determines the number of adipocytes seen after 9–10 d. Expression of *WNT5A*, *WNT5B* and at lower levels *WNT2B*, *WNT3* and *WNT11* and multiple isoforms of *FZD* are detected across datasets, potentially orchestrating signaling. Analysis of specific ligand–receptor pairs in non-induced progenitor cells and following adipogenic induction at single-cell resolution and in the context of cell localization and tissue architecture will be needed to provide further insights. It is important to note that Wnt ligands are also expressed in other cell types within the adipose tissue and therefore the local cellular microenvironment can play a role in normal or pathological development of adipose tissue through Wnt signaling (Extended Data Fig. 5f).

**Extended Data Fig. 6:**
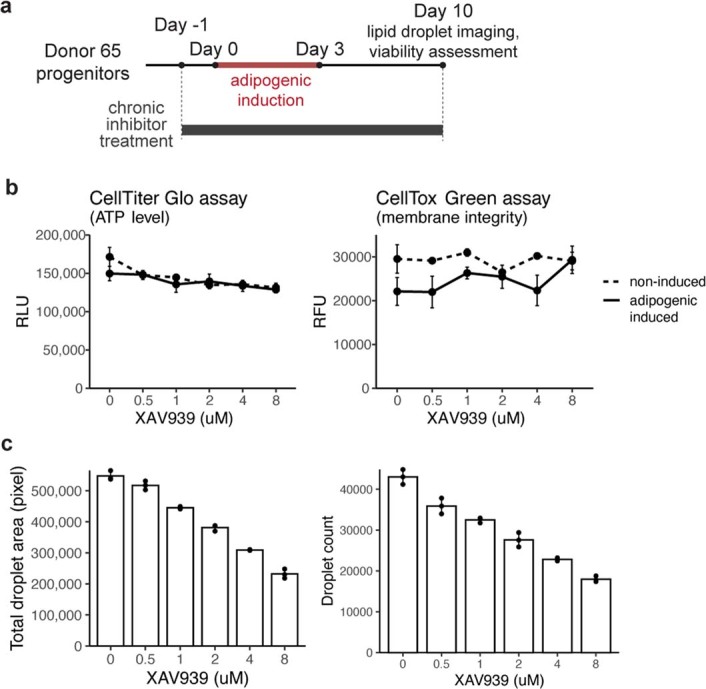
Long term Wnt antagonist treatment suppresses adipogenesis. **a**, Schematic of the adipogenesis assay with chronic Wnt inhibition. **b**, Viability assessment of 10-day adipogenic-induced cells under chronic exposure to different dosages of XAV939 (error bars = SD, *n* = 3). **c**, Lipid droplet quantification of 10-day adipogenic-induced cells under chronic exposure to different dosages of XAV939 (error bars = SD, *n* = 3).

It is important to note that Wnt signaling may also influence the properties of cells after they have undergone commitment to the adipocyte fate. In our studies, inhibition of tankyrase and thereby suppression of Wnt signaling early during adipogenic induction promotes adipogenesis, but chronic inhibition of tankyrase throughout 7 d of differentiation suppressed adipocyte development without inducing toxicity (Extended Data Fig. 6). A role for Wnt signaling after fate determination is also supported by the report of impaired lipogenesis in mice in which β-catenin excision was driven by *Adipoq*-Cre, which is by definition post-adipogenic determination^8^. The distinct mechanisms by which Wnt signaling controls fate determination early upon differentiation induction and supports adipocyte function post-fate commitment remain to be defined.

While complex, our finding of a mechanism for maintenance of adipocyte progenitor pool driven by Wnt signaling will help deconvolve mechanisms operating at different stages of adipose tissue development in diverse depots and under diverse physiological states Local Wnt levels may determine individual variation in adipose tissue distribution and its propensity toward hyperplasia or hypertrophy, which are associated with metabolic disease risk. Indeed, one of the first discovered genetic associations with type 2 diabetes was with the Wnt pathway gene *TCF7L2*, potentially acting on adipose tissue to confer enhanced risk^12,13,54,55^. In addition, adipose tissue surrounds various organs, such as the heart and blood vessels. Multipotent progenitor cells within adipose tissue may play a role in the repair of nearby organs and tissues, highlighting another potential link between the maintenance of a progenitor cell pool in adipose tissue and overall health throughout the lifespan.

## Methods

### Adipose tissue explants

Methods for the collection and the culture of adipose tissue explants were previously published^23^. In short, subcutaneous adipose tissues were donated from consented adult patients undergoing elective panniculectomy surgery at University of Massachusetts Medical Center (Extended Data Table 1). Individuals were recruited in a consecutive fashion without consideration of sex and samples were placed into culture within 1–6 h after tissue excision. The 200 explants of approximately 1 cm^3^ in size were embedded in Matrigel Matrix (cat. no. 356231, Corning) per 10-cm dish with EGM-2MV (cat. no. CC-3156,CC-4147, Lonza) medium supplementation. The progenitors in explants were allowed to grow for 14 d in Matrigel with fresh medium replacement every 2–3 d. After 14 d, progenitors in explants were recovered using dispase (cat. no. 354235, Corning) for 1 h followed by additional 14 min of trypsin-EDTA (cat. no. 15400-054, Gibco) and collagenase I (cat. no. LS004197, Worthington) and plated on standard tissue culture plate for expansion and cryopreservation.

### Lineage differentiation

Adipogenic differentiation was induced by providing confluent cells with DMEM (cat. no. 11995-065, Gibco) and 10% fetal bovine serum (FBS) (cat. no. 25-514, Genesee Scientific) supplemented with 0.5 mM 3-isobutyl-1-methylxanthine (cat. no. I5879, Sigma), 0.25 μm dexamethasone (cat. no. D1756, Sigma) and 5 μg ml^−1^ insulin (cat. no. 15500, Sigma). The induced cells underwent half medium replacement every 24 h for 3 d. After 3 d, cells were maintained in DMEM + 10% FBS with half medium replacement every 2–3 d until collection.

Chondrogenic differentiation was induced by providing confluent cells with DMEM + 10% FBS supplemented with 1 mM sodium pyruvate (cat. no. 11360-070, Gibco), 100 nM dexamethasone (cat. no. D1756, Sigma), 10 ng ml^−1^ Human TGF-β 1 recombinant protein (cat. no. PHG9204, Gibco) and 1 μg ml^−1^ l-ascorbic acid 2-phosphate (cat. no. A8960, Sigma). The induced cells underwent half medium replacement every 2–3 d until collection.

Osteogenic differentiation was induced by providing confluent cells with DMEM and 10% FBS supplemented with 10 mM sodium β-glycerolphosphate (cat. no. L03425, Alfa Aesar), 100 nM dexamethasone (cat. no. D1756, Sigma), 50 uM l-ascorbic acid 2-phosphate (cat. no. A8960, Sigma). The induced cells underwent half medium replacement every 2–3 d until collection.

Cells in the growth condition were cultured in EGM-2MV. Cells in the control condition were maintained in DMEM + 10% FBS. All cells underwent identical medium replacement to the experimental group during experiments.

### Lineage staining

Cells were washed with phosphate-buffered saline (PBS) and fixed with 10% formalin for 30 min at room temperature. Following fixation, cells were washed three times with double distilled water. To stain for adipocyte lipid droplets, cells were first incubated in 60% isopropanol for 5 min followed by staining with 2% Oil Red O (cat. no. 00625, Sigma) in 60% isopropanol for 10 min. For assessment of chondrogenesis, cells were incubated with 1% Alcian blue 8GX (cat. no. A5268, Sigma) in 2:3 acetic acid and ethanol solution in the dark for 30 min with gentle agitation. For assessment of osteogenesis, cells were incubated with 2% Alizarin red S staining solution (cat. no. 0223, ScienCell) in the dark for 30 min with gentle agitation. After each staining protocol, the staining solution was removed and cells were washed three times with double distilled water before imaging.

### Flow cytometry analysis

Mesenchymal cell surface marker expression of the three-dimensional culture-derived and SVF-derived progenitors were assessed using the MSC Characterization Antibody Panel (cat. no. 100-0354, Stemcell Technology) following manufacturer’s instruction.

### RNA extraction for bulk RNA-sequencing and qPCR

Cells in culture wells were washed with PBS before collection with TriPure TRIzol reagent (cat. no. 11 667 165 001, Roche). The cell-TRIzol mixtures were transferred to collection tubes and homogenized with Tissuelyser II (QIAGEN). Chloroform was added in a 1:5 ratio by volume and phase separation was performed. The RNA-containing layer was mixed with an equal volume of 100% isopropanol and incubated overnight at −20 °C for precipitation. RNA was pelleted and washed with 80% ethanol and resuspended in nuclease-free water. RNA concentration and purity were determined using a NanoDrop 2000 (Thermo Scientific). RNA for sequencing were sent to University of Massachusetts Medical School Molecular Biology Core Laboratory for fragment analysis.

### Bulk RNA-sequencing

Library preparation was performed using TruSeq Stranded mRNA Low-Throughput Sample Prep kit (cat. no. 20020594, Illumina) according to manufacturer’s instruction. The libraries were sequenced on the NextSeq 500 system (Illumina) using the NextSeq 500/550 High Output kits v2 (75 cycles; single-end sequencing; cat. no. FC-404-2005, Illumina). The FASTQ files were processed using the DolphinNext pipeline^56^ on the Massachusetts Green High Performance Computer Cluster (GHPCC). DolphinNext was configured to use RSEM for read mapping and transcript quantification^57^. Differentially expressed genes were identified using DESeq2 (ref. ^58^). Pathway analysis was performed using Gene Set Enrichment Analysis software with the MSigDB Gene Ontology biological process gene sets (http://www.gsea-msigdb.org/gsea/msigdb/annotate.jsp)^59^. Sequencing results have been deposited in the Gene Expression Omnibus (accession nos. GSE198275, GSE198481, GSE204847 and GSE204848).

### Single-cell RNA-sequencing

Single-cell library preparation was performed using Chromium Single Cell 3′ GEM Library & Gel Bead Kit v3 (cat. no. 1000092, 10x Genomics) according to manufacturer’s instruction. The libraries were sequenced on the NextSeq 500 system (Illumina) using the NextSeq 500/550 High Output kits v.2.5 (50 cycles; cat. no. FC-404-2005, Illumina). The sequencing outputs were processed using the CellRanger software v.3.1.0 on the GHPCC. Reads were mapped to human reference genome GRCh38 (Ensembl 93). Data analysis was performed using Seurat v.4.1.0 (ref. ^60^) within R v.4.0.2 environment. RNA velocity analysis was performed using the velocyto v.0.17 commend line tool and velocyto.R v.0.6 R package^61^. Sequencing results have been deposited in the Gene Expression Omnibus (accession no. GSE198482) and analysis scripts are available at https://github.com/zingery/mesenchymal-maintenance/.

### Quantitative PCR with reverse transcription

A total of 1 µg of RNA was reverse transcribed using the iScript cDNA Synthesis kit (cat. no. 1708891, Bio-Rad) according to manufacturer’s protocol. Quantitative PCRs with reverse transcription were prepared with iQTM SYBR Green Supermix (cat. no. 1708882, Bio-Rad) and were performed on a CFX Connect Real-Time PCR Detection System (Bio-Rad). qRT−PCR data were collected by Bio-Rad CFX Maestro v.2.3 Software. The *ADIPOQ* primers have the following sequences: 5′-TGC TGG GAG CTG TTC TAC TG-′3 forward and 5′-TAC TCC GGT TTC ACC GAT GTC-′3 reverse. The *MGP* primers have the following sequences: 5′-CAG CAG AGA TGG AGA GCT AAA G-′3 forward and 5′-GTC ATC ACA GGC TTC CCT ATT-′3 reverse. The *PLIN1* primers have the following sequences: 5′-ACC AGC AAG CCC AGA AGT C-′3 forward and 5′-CAT GGT CTG CAC GGT GTA TC-′3 reverse. The *FRZB* primers have the following sequences: 5′-GCC CTG GAA CAT GAC TAA GAT G-3′ forward and 5′-GTA CAT GGC ACA GAG GAA GAA G-′3 reverse. The *COMP* primers have the following sequences 5′-CCA ACT CAA GGC TGT GAA GTC-′3 forward and 5′-GGA CTT CTT GTC CTT CCA ACC-3′ reverse.

### Lipid droplet phase image acquisition and processing

Cells were imaged with LEICA DM 2500 LED inverted microscope equipped with a Leica MC120 HD digital camera. Fiji/ImageJ v.1.53c software was used to quantify lipid droplets. The images were converted from RGB to eight-bit, background subtracted, contrast enhanced, thresholded and binarized followed by circular particle analysis.

### Cell separation with Percoll density gradient

A Percoll step density gradient was prepared in a 15-ml conical tube with the Percoll solutions (cat. no. P4937, Sigma). Earlier experiments (Fig. 3a–c) were performed in Percoll densities of 1.010 g ml^−1^, 1.020 g ml^−1^, 1.030 g ml^−1^ and 1.040 g ml^−1^, whereas later experiments were performed in Percoll densities of 1.010 g ml^−1^, 1.020 g ml^−1^ and 1.030 g ml^−1^ to permit HD cells to be collected from the cell pellet after centrifugation. The 7-d adipogenic-induced cell populations were lifted with StemPro Accutase (cat. no. A1110501, Thermo Fisher). Lifted cells were pelleted and resuspended in 1.010 g ml^−1^ Percoll solution and loaded onto the top of the prepared Percoll gradient, followed by centrifugation at 1,000*g* for 30 min at room temperature. Cell fractions were observed by eye and each resulting fraction was pipetted into new conical tubes for further processing.

### Ligand–receptor analysis

First, we identified genes expressed in cluster 3 and cluster 5 from the single-cell dataset presented in Fig. 2. A gene was considered as expressed within a cell cluster if average normalized counts ≥ 0.5. We then queried the putative or literature supported ligand–receptor pairs obtained from Ramilowski et al. to identify gene pairs expressed in both clusters^62^.

### Cell viability assays

CellTiter-Glo 2.0 cell viability assay (cat. no. G9243, Promega) and CellTox Green cytotoxicity assay (cat. no. G8742, Promega) were performed according to manufacturer’s instruction and the fluorescence and luminescence signals were measured using a Safire 2 microplate reader (Tecan).

### Small molecule inhibitors

Tankyrase inhibitor XAV939 (cat. no. HY-15147) and GSK3 inhibitor CHIR99021 (cat. no. HY-10182) were obtained from MedChemExpress.

### Super TopFlash reporter assay

The 7TFC STF luciferase lentiviral vector was from Roel Nusse (Addgene plasmid 24307)^31^. Lentiviruses were produced using Lenti-X 293T Cell Line from Takara (cat. no. 632180) following the standard virus packaging protocol. Progenitor cells were infected with the packaged 7TFC vector before induction toward adipogenesis. Cell lysates were collected for relative luciferase activity measurement with the Luciferase Assay System (cat. no. E1500, Promega). Luminescence signals were measured using a Safire 2 microplate reader (Tecan). Measured luciferase signals were normalized by total protein quantity measured using Pierce BCA Protein assay (cat. no. 23227, Thermo Scientific).

### Genetic perturbation of the canonical Wnt pathway

The E[beta]C and EdTC lentiviral vectors for stable β-catenin and dominant-negative *TCF* overexpression were from R. Nusse (Addgene plasmid 24312, 24310)^31^. Lentiviruses were produced using Lenti-X 293T Cell Line from Takara (cat. no. 632180) following the standard virus packaging protocol. Progenitor cells were infected with the packaged vectors before induction toward adipogenesis. Cells were differentiated for 7 d and assessed by fluorescence microscopy after fixation and staining. Cells were washed with PBS and fixed with 4% paraformaldehyde for 30 min at room temperature, followed by lipid and nuclei staining with HCS LipidTOX Green Neutral Lipid Stain (cat. no. H34475, Invitrogen) at 1:200 dilution and Hoechst 33342 (cat. no. 62249, Thermo Scientific) at 1:20,000 dilution for 30 min at room temperature. mCherry reporter and stain signals were visualized and captured using a Zeiss Axiovert 200 M Fluorescence microscope. ImageJ (FIJI) was used for image processing and normalization.

### Human adipose tissue development in immunocompromised mice

Methods for the collection and culture of adipose tissue explants were previously published^33^. In brief, ~10^7^ passage 3 human adipose tissue derived progenitor cells were thawed into 150-mm plate and after 72 h they were split 1:2 at a dense seeding density of ~8 × 10^6^ cells per 150-mm plate. Upon confluence (approximately 72 h after plating), adipogenic differentiation was induced and allowed to differentiate for 10 d. Single-cell suspensions of differentiated adipocytes were obtained by incubation for 10 min in a mixture of trypsin-EDTA (cat. no. 15400-054, Gibco) and 0.5 mg ml^−1^ collagenase (cat. no. 354235, Corning). Proteases were quenched by dilution into culture medium and cells pelleted by centrifugation at 500*g* for 10 min. The medium layer between floating and pelleted cells was removed and remaining cells were brought to 1 ml total volume with cold PBS, placed on ice and mixed with an equal volume of cold Matrigel (cat. no. 356231, Corning). Then, 0.5-ml aliquots of the cell suspension were placed in 1-ml syringes on ice and injected subcutaneously into each flank of immunodeficient male nude mice, 8–10 weeks old (Nu/J Jackson labs stock no. 002019) using a 20G needle. At the end of the 8-week time point, all the animals were killed and tissues were collected for analysis. All procedures were performed in accordance with the University of Massachusetts Medical School’s Institutional Animal Care and use Committee protocol PROTO202100015. The implant sequencing results were aligned to both the human (hg38) and the mouse (mm10) genome. The resulting alignments were processed using the R-package XenofilteR to classify reads as either of human or mouse origin^63^.

### RNAscope

*FABP4* and *MGP* mRNAs were visualized using the RNAscope Multiplex Fluorescent Detection kit v.2 (cat. no. 323100, ACD Bio). RNAscope was performed using target probes to *FABP4* (cat. no. 470641-C2, ACD Bio) and *MGP* (cat. no. 586051, ACD Bio) and labeled with Opal Fluorophore Reagents (cat. no. FP1487001KT and FP1488001KT, Akoya Biosciences) according to the manufacturer’s protocol. Signals were visualized and captured using a Zeiss Axiovert 200M Fluorescence microscope. ImageJ (Fiji) was used for image processing and normalization. All images analyzed were filtered to remove background using identical parameters. Masks of images in each channel (for *MGP* or for *FABP4* signals) were generated and overlaid on the original image (for example *MGP* image) and on the corresponding *FABP* image to obtain the intensity value for each signal in each cell and values were plotted.

### Statistics and reproducibility

All results presented in this study with the exception RNA-seq profiling and RNAscope experiment have been repeated with cells from independent donors. No statistical method was used to predetermine sample size. No data were excluded from the analyses unless stated otherwise. All statistical analyses were performed using R v.4.0.2 or GraphPad Prism v.9.5.1. All error bars represent s.d.

### Reporting summary

Further information on research design is available in the Nature Portfolio Reporting Summary linked to this article.

### Supplementary information

Supplementary Information Supplementary Table 1 and Supplementary Fig. 1.

Reporting Summary

Supplementary Table 2 Ligand-receptor interactions among cells along the differentiation trajectory.

### Source data

Source Data Fig. 3 Statistical Source Data.

Source Data Fig. 4 Statistical Source Data.

Source Data Fig. 5 Statistical Source Data.

Source Data Extended Data Fig. 1 Statistical Source Data.

Source Data Extended Data Fig. 5 Statistical Source Data.

## Figures and Tables

**Extended Data Table 1:**
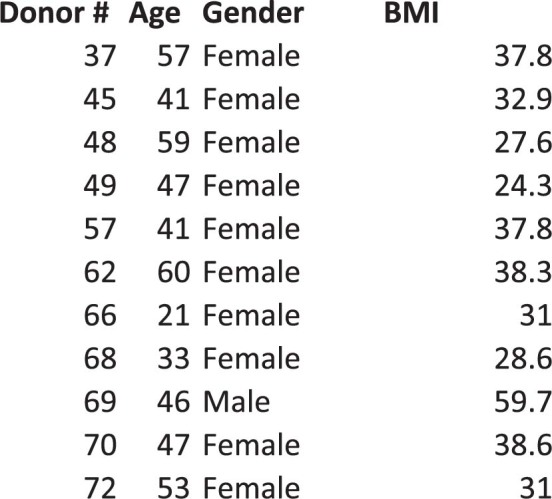
Human adipose tissue donor demographics

BMI, age and gender information for all human adipose tissue donor.

## Data Availability

Single-cell RNA expression data are deposited in the Single Cell Portal (study no. SCP1027). Raw sequencing reads and processed count data for single and bulk RNA-seq have been deposited in the Gene Expression Omnibus (GSE198482, GSE198275, GSE198481, GSE204847 and GSE204848). Single-cell sequencing reads were mapped to human reference genome GRCh38 and all bulk RNA-seq results were mapped to human reference genome GRCh37 (hg19). Source data are provided with this paper.

## Code availability

Code used in this study for the analysis of the single-cell RNA-seq data is available at https://github.com/zingery/mesenchymal-maintenance/.
