# Supplementary material for: Wnt signaling preserves progenitor cell multipotency during adipose tissue development
Source: Nat Metab. 2023 Jun 19;5(6):1014–28. doi: 10.1038/s42255-023-00813-y (PMC10290956; doi:10.1038/s42255-023-00813-y)

nature metabolism

Article https://doi.org/10.1038/s42255-023-00813-y

Wnt signaling preserves progenitor cell multipotency during adipose tissue development

In the format provided by the authors and unedited

Supplementary Table 1. Flow cytometry gating strategy

**Laser**	Violet-405nm	Blue-488nm		Green-532nm	Red-633nm
**Fluorochrome**	DAPI	FITC	PerCP/Cy5.5	PE	APC
**MSC Cells**	Live/dead	CD90 (5E10)	CD45 (H130)	CD73 (AD2)	CD105 (SN6H)

Supplementary Fig. 1. for Extended Data Fig. 1c,d. Flow cytometry gating strategy

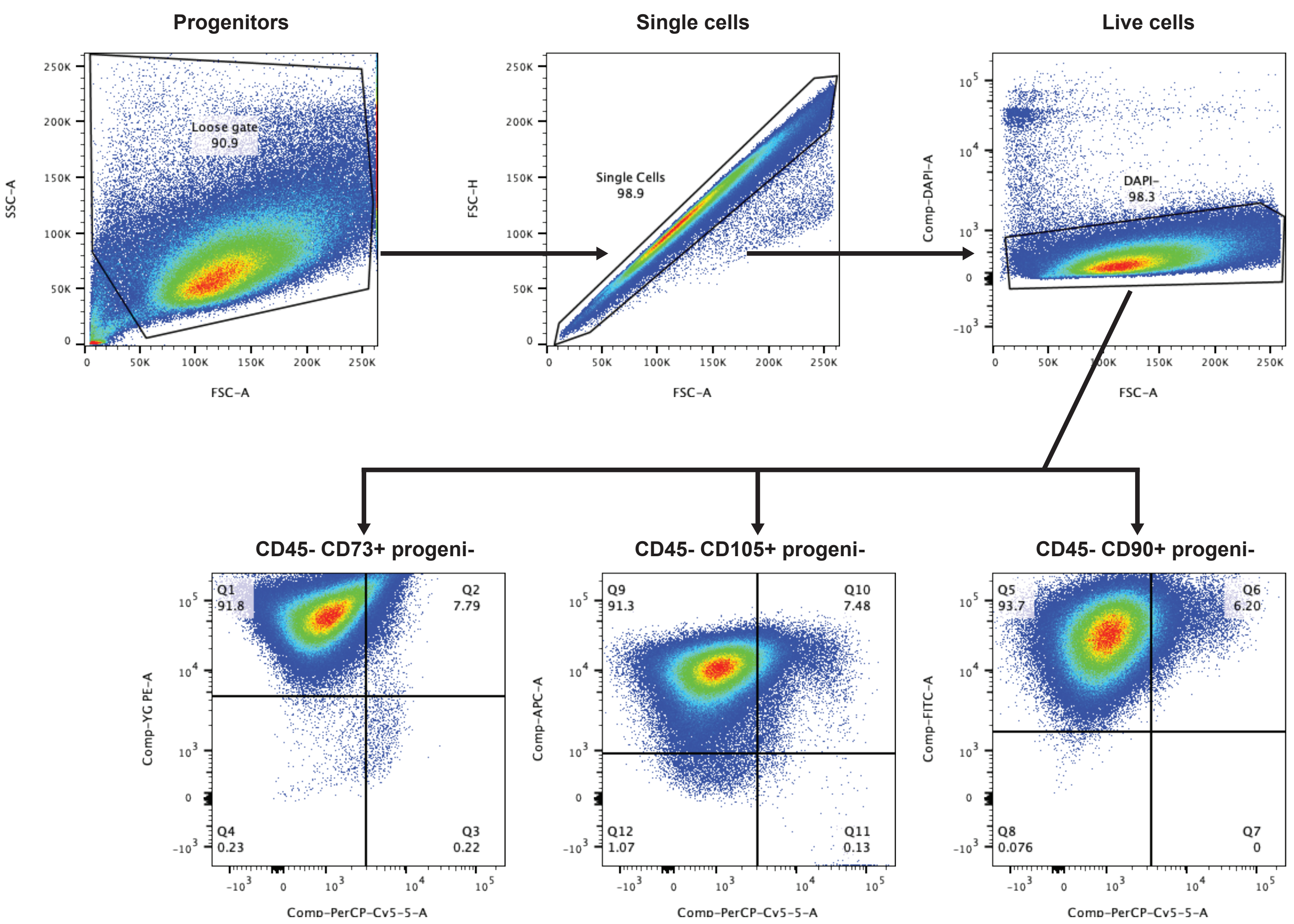

Supplement: Supplementary file 1 — Supplementary Table 1 and Supplementary Fig. 1. [file 42255_2023_813_MOESM1_ESM.pdf]
