# Supplementary material for: Wnt signaling preserves progenitor cell multipotency during adipose tissue development
Source: Nat Metab. 2023 Jun 19;5(6):1014–28. doi: 10.1038/s42255-023-00813-y (PMC10290956; doi:10.1038/s42255-023-00813-y)
Supplement: Supplementary file 2 — Reporting Summary [file 42255_2023_813_MOESM2_ESM.pdf]

nature portfolio

Corresponding author(s): Silvia Corvera
Last updated by author(s): April 6 0,2023

Reporting Summary

Nature Portfolio wishes to improve the reproducibility of the work that we publish. This form provides structure for consistency and transparency in reporting. For further information on Nature Portfolio policies, see our Editorial Policies and the Editorial Policy Checklist.

Statistics

For all statistical analyses, confirm that the following items are present in the figure legend, table legend, main text, or Methods section.

n/a	Confirmed	
☐	☒	The exact sample size (*n*) for each experimental group/condition, given as a discrete number and unit of measurement
☐	☒	A statement on whether measurements were taken from distinct samples or whether the same sample was measured repeatedly
☐	☒	The statistical test(s) used AND whether they are one- or two-sided<br>*Only common tests should be described solely by name; describe more complex techniques in the Methods section.*
☐	☒	A description of all covariates tested
☐	☒	A description of any assumptions or corrections, such as tests of normality and adjustment for multiple comparisons
☐	☒	A full description of the statistical parameters including central tendency (e.g. means) or other basic estimates (e.g. regression coefficient) AND variation (e.g. standard deviation) or associated estimates of uncertainty (e.g. confidence intervals)
☐	☒	For null hypothesis testing, the test statistic (e.g. *F*, *t*, *r*) with confidence intervals, effect sizes, degrees of freedom and *P* value noted<br>*Give P values as exact values whenever suitable.*
☒	☐	For Bayesian analysis, information on the choice of priors and Markov chain Monte Carlo settings
☒	☐	For hierarchical and complex designs, identification of the appropriate level for tests and full reporting of outcomes
☒	☐	Estimates of effect sizes (e.g. Cohen's *d*, Pearson's *r*), indicating how they were calculated

Our web collection on statistics for biologists contains articles on many of the points above.

Software and code

Policy information about availability of computer code

Data collection

qRT-PCR data was collected by Bio-RAD CFX Maestro Software. Fluorescence and luminescence assay results were collected on a Safire 2 microplate reader (Tecan) via the XFlour software.

Data analysis

All statistics presented in this study were performed using R version 4.0.2.

Bulk RNA-sequencing
The sequencing output FASTQ files were processed using the DolphinNext pipeline on the Massachusetts Green High Performance Computer Cluster (GHPCC). DolphinNext was configured to use RSEM for read mapping and transcript quantification. Differential expression analysis of bulk RNA-sequencing results were performed using DESeq2 v1.28.1.

Single-cell RNA-sequencing
The sequencing outputs were processed using the CellRanger software v3.1.0 on the Massachusetts Green High Performance Computer Cluster (GHPCC), Reads were mapped to human reference genome GRCh38 (Ensembl 93). Data analysis was performed using Seurat v4.1.0 40 within R version 4.0.2 environment. RNA velocity analysis was performed using the velocyto v0.17 commend line tool and velocyto.R v0.6.

For manuscripts utilizing custom algorithms or software that are central to the research but not yet described in published literature, software must be made available to editors and reviewers. We strongly encourage code deposition in a community repository (e.g. GitHub). See the Nature Portfolio guidelines for submitting code & software for further information.

Data

Policy information about availability of data

All manuscripts must include a data availability statement. This statement should provide the following information, where applicable:

- Accession codes, unique identifiers, or web links for publicly available datasets
- A description of any restrictions on data availability
- For clinical datasets or third party data, please ensure that the statement adheres to our policy

All relevant data supporting the findings of this study are available within the manuscript and the supplemental information file.
Bulk and single-cell RNA-sequencing results were submitted to GEO (accession number: GSE198275, GSE198481, GSE204847, GSE204848, GSE198482). In additional to transcriptomic datasets generated by our lab, we analyzed the single cell atlas of human white adipose tissue made available by Emont, et al. (https://singlecell.broadinstitute.org/single_cell/study/SCP1376/a-single-cell-atlas-of-human-and-mouse-white-adipose-tissue).

Human research participants

Policy information about studies involving human research participants and Sex and Gender in Research.

Reporting on sex and gender	Cells derived from human tissue used in this study were selected from sequential donors without consideration of gender or sex. The 7 donor-derived cells used n this study were obtained from female donors.
Population characteristics	This study does not constitute human research. Participants were not recruited for the purpose of this study. Donor-derived cells used were from consecutive donors and were used for the experiments described without consideration of age, sex or other individual variations.
Recruitment	Participants were not specifically recruited for the purpose of this study. This study was conducted with donor-derived cells obtained from consecutive donors and were used without consideration of age, sex or other individual human variation.
Ethics oversight	Human tissue collection was conducted in accordance with the UMass Chan Institutional Review Board ID 14734_13.

Sample size	Sample size of experiments is based on effect sizes assessed from preliminary experiment and the available number of the human donor-derived primary cells.
Data exclusions	No data was excluded
Replication	All experiments, with the exception of the transcriptomic profiling studies, were repeated at least two times with cells derived from different donors. All repeated experiments confirmed the original findings.
Randomization	Selection of donor cells were based on cell availability. No selection criteria was applied.
Blinding	Blinding was not conducted given that this study was not a randomized control format. To minimize bias, results from each experimennt were independently analyzed by multiple operators. Immunofluorescence images were acquired by different operators.

Reporting for specific materials, systems and methods

We require information from authors about some types of materials, experimental systems and methods used in many studies. Here, indicate whether each material, system or method listed is relevant to your study. If you are not sure if a list item applies to your research, read the appropriate section before selecting a response.

Materials & experimental systems

n/a	Involved in the study
☒	☐ Antibodies
☐	☒ Eukaryotic cell lines
☒	☐ Palaeontology and archaeology
☐	☒ Animals and other organisms
☒	☐ Clinical data
☒	☐ Dual use research of concern

Methods

n/a	Involved in the study
☒	☐ ChIP-seq
☒	☐ Flow cytometry
☒	☐ MRI-based neuroimaging

Eukaryotic cell lines

Policy information about cell lines and Sex and Gender in Research

Cell line source(s)	Adult human adipose-tissue derived primary mesenchymal progenitors; Lenti-X™ 293Tcells
Authentication	Cells used in this study were non-transformed primary progenitors cells derived from individual donors, and were subjected to standard quality control checks, including growth rate and multipotency. No additional validation was performed. Lenti-X™ 293Tcells were obtained from Takara (Cat# 632180)
Mycoplasma contamination	Not tested.
Commonly misidentified lines (See ICLAC register)	No commonly misidentified line were used.

Animals and other research organisms

Policy information about studies involving animals; ARRIVE guidelines recommended for reporting animal research, and Sex and Gender in Research

Laboratory animals	male nude mice, 8-10 weeks old, (Nu/J Jackson labs stock no: 002019)
Wild animals	No wild animals were used in this study.
Reporting on sex	Mice were used as hosts to harbor developing adipose tissue from human progenitor cells derived from female donors. The sex of the mouse was not considered a relevant variable.
Field-collected samples	No field-collected samples were used in this study.
Ethics oversight	All procedures were performed in accordance with the University of Massachusetts Medical School's Institutional Animal Care and use Committee protocol PROTO202100015.

Note that full information on the approval of the study protocol must also be provided in the manuscript.
